# Isolation and characterization of Sporomusa carbonis sp. nov.: a carboxydotrophic hydrogenogen in the genus of Sporomusa isolated from a charcoal-burning pile

**DOI:** 10.1099/ijsem.0.006677

**Published:** 2025-04-16

**Authors:** Tim Böer, Florian P. Rosenbaum, Lena Eysell, Volker Müller, Rolf Daniel, Anja Poehlein

**Affiliations:** 1 Genomic and Applied Microbiology and Göttingen Genomics Laboratory, Institute of Microbiology and Genetics, Georg-August-University, Göttingen, Germany; 2 Department of Molecular Microbiology & Bioenergetics, Institute of Molecular Biosciences, Johann Wolfgang Goethe University, Frankfurt, Germany

**Keywords:** acetogen, carbon monoxide, carboxydotrophic hydrogenogen, *Sporomusa*, *Sporomusaceae*, Wood–Ljungdahl pathway

## Abstract

A Gram-negative bacterial strain, designated ACPt^T^, was isolated from the top of the covering soil of an active charcoal-burning pile. The cells of ACPt^T^ were strictly anaerobic, rod-shaped and grew optimally at 40 °C and pH 7. The substrates ribose, glucose, sucrose, raffinose, melezitose, pyruvate, vanillate, syringate, methanol and CO were utilized for growth. Phylogenomic analysis of the 4.1 Mb genome showed that strain ACPt^T^ represented a novel species of the genus *Sporomusa*. The most closely related species to ACPt^T^ was *Sporomusa malonica,* with an average amino acid identity of 80.1%. The genome of ACPt^T^ encoded cytochromes, ubiquinones, the Wood–Ljungdahl gene cluster and an Rnf complex, which were identified as common features of all *Sporomusa* type strains. However, strain ACPt^T^ did not ferment H_2_+CO_2_ via acetogenesis as other *Sporomusa* species but employed the metabolism of a carboxydotrophic hydrogenogen, converting CO to H_2_+CO_2_. Based on the genomic, morphological and physiological features presented in this study, strain ACPt^T^ is proposed as a novel species in the genus *Sporomusa,* with the name *Sporomusa carbonis* sp. nov. (DSM 116159^T^ and CCOS 2105^T^).

## Data Summary

The authors confirm that all supporting data, code and protocols have been provided within the article or through supplementary data files.

## Introduction

The genus *Sporomusa* was formed in 1984 by the description of *Sporomusa ovata,* isolated from sugar beet leaf silage, and *Sporomusa sphaeroides,* isolated from mud of the Leine river [1]. Hitherto, seven other *Sporomusa* species were validly published under the ICNP, comprising *Sporomusa paucivorans* [2], *Sporomusa termitida* [3], *Sporomusa acidovorans* [4], *Sporomusa malonica* [5], *Sporomusa silvacetica* [6], *Sporomusa aerivorans* [7] and *Sporomusa rhizae* [8]. Bacteria of the genus *Sporomusa* are morphologically characterized by their motile, curved rod-shaped and endospore-forming cells. Furthermore, *Sporomusa* cells stain Gram-negative and contain a multilayered cell wall [1]. Representatives of the genus *Sporomusa* are well known for utilizing H_2_+CO_2_, alcohols (such as methanol, propanol and butanol), *N*-methylated compounds (such as sarcosine and betaine) and methoxylated monoaromates (such as vanillate and syringate) for growth [9]. H_2_+CO_2_ is converted via acetogenesis using the Wood–Ljungdahl pathway (WLP), while growth on alcohols, *N*-methylated and methoxylated compounds occurs by transferring methyl groups via a methyltransferase system and subsequent feeding of the methyl groups into the methyl-branch of the WLP [10]. Autotrophic growth on CO has only been reported for *S. ovata* [11] and *S. termitida* [3]. Energy conservation during acetogenesis in the mesophilic genus *Sporomusa* is achieved by building a chemiosmotic H^+^-gradient using an Rnf complex, in contrast to thermophilic acetogens utilizing an Ech complex. The H^+^-gradient is subsequently utilized by an ATP synthase for the phosphorylation of ADP to ATP [12]. The genus *Sporomusa* comprises exclusively acetogenic and mesophilic strains containing both cytochromes b/c and ubiquinones. The presence of cytochromes and quinones is a rare trait of acetogens and, besides *Sporomusa* members, is only present in acetogens of the thermophilic *Moorella* genus. Cytochromes and quinones were hypothesized to offer alternative routes for energy conservation during acetogenesis using the HdrABC-MvhD or Fix complex [13,14]. Recently, *S. ovata* was shown to employ a three-subunit *Sporomusa*-type Nfn transhydrogenase (Stn) instead of the two-subunit Nfn transhydrogenase used by other acetogens for linking the redox pool of NADH and reduced ferredoxin during autotrophy to the cellular redox pool (NADPH) [15]. Biotechnological applications of bacteria from the genus *Sporomusa* have focused on the CO_2_-based production of bulk chemicals and biofuels via microbial electrosynthesis [16,21] or a bacterial CdS biohybrid system [22]. *S. ovata* is particularly suitable for microbial electrosynthesis, as it exhibited some of the lowest H_2_-threshold described for acetogens [23]. Another biotechnological application is the transformation of cheap waste substrates such as methanol, as an industrial feedstock for the production of biocommodities [24,25]. For the fermentation of CO-rich industrial waste gases, acetogens can be grown in co-culture with a carboxydotrophic hydrogenogenic bacterium, initially converting CO with the water–gas shift reaction to H_2_+CO_2_. The produced H_2_+CO_2_ is subsequently fermented by an acetogen to acetate. For instance, the conversion of CO and acetate production of *S. ovata* could be significantly increased when grown in co-culture with *Citrobacter amalonaticus* [26]. The recent implementation of genetic tools for *S. ovata* has further paved the way for the utilization of *Sporomusa* members as industrial platform organisms [27]. All these applications of *Sporomusa* as a biocatalyst offer a renewable and sustainable route for the production of biocommodities while simultaneously fixing the greenhouse gas CO_2_. Here, we present the description of the first carboxydotrophic hydrogenogen isolated from the genus *Sporomusa* and provide an analysis of high-quality genomes of *Sporomusa* type strains, focusing on genes and complexes potentially employed during lithotrophic growth.

## Methods

### Enrichment, isolation and cultivation

A sample from the top of the covering soil of an active burning charcoal pile in Hasselfelde, Germany (51° 43′ 18.6″ N 10° 53′ 48.6″ E) was taken in October 2021. The sample (1 g) was dissolved (50% w/v) in PBS (NaCl, 8 g l^−1^; KCl, 0.2 g l^−1^; Na_2_HPO_4_, 1.42 g l^−1^; KH_2_PO_4_, 0.24 g l^−1^; pH=7.4) by shaking at 50 r.p.m. for 1 h on a horizontal shaker (Adolf Kühner, Birsfelden, Swiss). Subsequently, the solution was pasteurized at 80 °C for 10 min, and 20 µl of the solution was used for the inoculation of enrichment cultures. The enrichment was performed in sterile anoxic Hungate tubes filled with 10 ml of a modified DSM 311 c minimal medium, substituting sulphate salts with chloride salts, reducing yeast extract to 0.05 g l^−1^ and omitting fructose and peptone. Syringate (5 mM) was added to the media as a substrate prior to autoclaving. The enrichment culture was incubated at 35 °C, and samples were taken after 96 and 264 h for cryoconservation (700 µl) by mixing the culture with 300 µl of glycerol (50%). Isolation was performed in the DSM 311 c minimal medium from the cryo stock taken after 264 h by inoculating two subsequent serial dilutions of cultures in media supplemented with methanol (300 mM). The highest dilution showing growth was used to inoculate the subsequent dilution series. After isolation, the isolate was routinely cultivated in the modified DSM 311 c medium of the enrichment cultures but as a complex medium with yeast extract (2 g l^−1^) and peptone (2 g l^−1^). d-glucose (30 mM) was added as a substrate from a sterile anoxic stock solution after media autoclaving. Reference *Sporomusa* strains were grown in the modified DSM 311 c complex medium inoculated from lyophilized cultures obtained from the German Collection of Microorganisms and Cell Cultures (DSMZ, Braunschweig, Germany). *S. acidovorans*, *S. aerivorans*, *S. paucivorans* and *S. rhizae* were grown with methanol (300 mM), while *S. silvacetica* and *S. malonica* were grown with d-fructose (30 mM). Both substrates were added from sterile anoxic stock solutions after media autoclaving.

### Physiological and morphological characterization

Temperature, pH and NaCl optima were determined by cultivation in the modified DSM 311 c complex medium supplemented with d-glucose (30 mM) inoculated to a starting OD_600_ of 0.01. Growth was measured as OD at 600 nm in Hungate tubes in triplicates after 48 h of incubation using the spectrophotometer WPA S800 (Biochrom, Berlin, Germany). The temperature optimum was investigated by incubation at temperatures ranging from 10 to 55 °C in 5 °C intervals. NaCl and pH optima were investigated at the respective temperature optima of the strains. NaCl tolerance was investigated with added NaCl concentrations of 0, 0.1, 1, 2, 3, 4 and 5%. The pH optimum was investigated by setting the pH to 5.5 to 9 in 0.5 intervals using HCl/NaOH. Gram staining was carried out with the Claus protocol [28]. Substrate utilization was evaluated by cultivation in Hungate tubes with the modified DSM 311 c minimal medium, adding substrates from sterile anoxic stock solutions in a final concentration of 30 mM. Vanillate (5 mM) and syringate (5 mM) were added to the media before autoclaving. Growth was measured from triplicate cultures after 48 h of incubation at the temperature optimum of the respective strain. Substrates not being used in the initial 48 h of incubation were continuously assessed for 2 weeks before substrate utilization was considered negative. Growth with H_2_+CO_2_ (66%/33%) and N_2_+CO (50%/50%) was tested by cultivation in 500 ml Afnor flasks with 50 ml of the modified DSM 311 c complex medium adding a final overpressure of 2 bar and horizontal incubation at 40 °C. Resting cell experiments with H_2_+CO_2_ and CO were performed at 40 °C, as described in [29]. The cellular fatty acid profile was determined by the identification service provided by the DSMZ. Cell morphology was investigated with a Jeol 1011 transmission electron microscope (Georgia Electron Microscopy, Freising, Germany) and negatively stained cells. Negative cell staining was achieved by mixing 5 µl of cell suspension with 5 µl of phospho-tungstic acid solution (0.5% w/v). The mixture was transferred to a vaporized carbon mica and subsequently placed on a copper-coated grid (PLANO GmbH, Marburg, Germany).

### Genome sequencing, assembly and analysis

DNA isolation and subsequent Illumina sequencing were performed, as described in [30]. For Nanopore sequencing, high-molecular-weight DNA was isolated from a separately cultivated batch with the Monarch Genomic DNA Purification Kit (New England Biolabs, Frankfurt, Germany), and library preparation was performed with 1.5 µg using the Ligation Sequencing Kit 1D (SQK-NBD114.24) and the Native Barcode Expansion Kit (EXP-NBD104) as recommended by the manufacturer (Oxford Nanopore Technologies, Oxford, UK). Nanopore sequencing was conducted for 72 h employing the MinION device Mk1B, the SpotON Flow Cell R10.4.1 and the MinKNOW software (v23.4.6), following the instructions of the manufacturer (Oxford Nanopore Technologies). Demultiplexing and base calling of Nanopore sequencing data were performed with the Dorado (v6.5.7) software in SUP mode. Long-read (Nanopore reads) *de novo* genome assemblies were performed with trycycler, as outlined by Wick *et al*. [31]. Quality control and adapter trimming of paired-end Illumina sequences was performed with Fastp (v0.23.4) [32] and Trimmomatic (v0.39; LEADING: 3, TRAILING: 3, SLIDINGWINDOW:4 : 15, MINLEN:50) [33]. Adapter trimming of Nanopore sequencing data was performed with Porechop (v0.2.4), sequences were subsequently quality filtered with Filtlong (v0.2.1) to a minimal read length of 1 kb and 5% of the sequences with the worst quality score were discarded. Filtered Nanopore reads were subsampled 24 times with Trycycler (v0.5.4) [31], and eight subsets each were used as input for the assemblers Flye (v2.9.2) [34], Canu (v2.2) [35] and Raven (v1.8.3) [36]. Assemblies were combined to a single consensus sequence with Trycycler, and the consensus sequence was polished with the full Nanopore data using Medaka (v1.10.0) and finally polished with the processed short-read sequences using Polypolish (v0.5.0) [37]. Genomes were reorientated manually to start with the *dnaA* gene. Genome annotations were performed with Prokka (v1.14.6) [38], and quality assessment of the final genome assemblies was conducted with CheckM2 (v1.0.2) [39]. Visualization of gene clusters was performed with clinker (v.0.0.28) [40], with genes showing a protein sequence identity of ≥50% being assigned the same colour. Phylogenomic analysis was carried out by determining the average nucleotide identity (ANI) using orthoANI (v0.5.0) [41], digital DNA–DNA hybridization values (dDDH) of the formula *d*_4_ using the Genome to Genome Distance Calculator (v3.0) [42], the percentage of conserved proteins (POCP) using the POCP pipeline (v2.3.2) [43] based on the method described by Quin *et al*. [44] and the average amino acid identity (AAI) using ezAAI (v1.2.3) [45]. The whole-genome sequence-based and 16S rDNA gene sequence-based phylograms were obtained by using the Type (Strain) Genome Server (TYGS) [46]. The core/pan-genome of the *Sporomusa* genus and strain-specific unique orthologous groups (OGs) were determined with Proteinortho (v6.3.0) [47], applying an identity threshold of ≥50% and an e-value of ≤1e^−10^. Unique OGs were visualized in R with the ggplot2 package (v3.4.1) [48]. Multilocus sequence analysis (MLSA) of the *Sporomusa* and *Methylomusa* type strains was conducted by obtaining singletons with Proteinortho (v6.3.0) using the same parameters as for the core/pan-genome, but excluding duplicated genes. Amino acid sequences of singletons were aligned with muscle (v3.8.31) [49], concatenated and unalignable regions were filtered out with Gblocks (v0.91b) [50]. Randomized Axelerated Maximum Likelihood (RAxML) (v8.2.12) [51] was used to infer the phylogenetic tree, which was subsequently visualized using FigTree (v1.4.4). The 16S rRNA gene sequence was amplified with a DreamTaq polymerase using genomic DNA as the template and the primers 08F (5′-AGAGTTTGATCCTGGC-3′) and 1504R (5′-TACCTTGTTACGACTT-3′) following the standard instructions of the manufacturer (Thermo Fisher Scientific, Waltham, MA, USA). The PCR product was purified using the MasterPure Complete DNA and RNA Purification Kit (Epicentre, Madison, USA) and Sanger sequenced by Seqlab (Microsynth Seqlab GmbH, Göttingen, Germany).

## Results and discussion

### Genomic and phylogenomic analysis

The genome assembly and annotation data for the *Sporomusa* genomes are summarized in Table 1. Genome sizes ranged from 4.1 to 6.5 Mb and average G+C content from 43 to 49.1 mol%. With 4.1 Mb, the genome size of isolate ACPt^T^ was considerably smaller than the reference *Sporomusa* genomes (4.8–6.5 Mb) and the *Methylomusa anaerophila* genome (4.8 Mb). With an average G+C content of 46.2 mol%, the genome of strain ACPt^T^ fell within the average G+C content of other *Sporomusa* genomes ranging from 43.0 to 49.1 mol% and was similar to the average G+C content of the *M. anaerophila* genome with 46.6 mol%. Correlating with the genome size, the lowest number of putative genes was encoded by the genome of the ACPt^T^ isolate (4148), while the highest number of genes was encoded by *S. aerivorans* (6303).

**Table 1.** Genome and annotation data of the novel ACPt^T^ strain, the *Sporomusa* type strains of *S. malonica*, *S. aerivorans*, *S. termitida*, *S. paucivorans*, *S. rhizae*, *S. sphaeroides*, *S. ovata*, *S. acidovorans*, * S. silvacetica* and the type strain *M. anaerophila*

	ACPtT*	S. malonica*	S. aerivorans*	*S. termitida*†	S. paucivorans*	S. rhizae*	*S. sphaeroides*‡	*S. ovata*‡	S. acidovorans*	S. silvacetica*	*M. anaerophila*§
Culture collection identifier	DSM 116159^T^ CCOS 2105^T^	DSM 5090^T^	DSM 13326^T^	DSM 4440^T^	DSM 3697^T^	DSM 16652^T^	DSM 2875^T^	DSM 2662^T^	DSM 3132^T^	DSM 10669^T^	JCM 31821^T^
Genome size (bp)	4 144 804	5 312 210	6 496 238	5 184 890	4 787 310	5 829 736	4 956 256	5 433 971	5 955 502	6 046 356	4 781 198
Plasmid size (bp)	–	–	15 390	131 118	–	–	59 087	–	–	–	–
G+C content (mol%)	46.2	44.6	46.7	49.1	47.3	43.3	47.2	43.3	44.7	43.0	46.6
CheckM2 completeness/contamination scores (%)	100/2.90	100/5.91	100/12.26	–	99.97/1.53	100/9.66	99.99/1.19	100/7.57	100/6.45	100/6.41	–
Genes	4148	5118	6303	4872	4483	5495	4656	5314	5750	5913	4268
CDS	4022	4955	6136	4735	4346	5324	4511	5112	5604	5757	4183
Functional proteins	2171	2757	3255	3659	2551	2965	2596	3609	3056	3015	3083
Hypothetical proteins	1851	2198	2881	1076	1795	2359	1915	1503	2548	2742	1100
rRNA (5S, 16S, 23S)	5, 11, 10	18, 16, 14	10, 14, 12	9, 12, 10	10, 11, 10	13, 14, 12	10, 12, 11	15, 15, 12	9, 11, 10	13, 14, 12	4, 4, 3
tRNAs	99	114	130	105	105	131	111	160	115	116	73
tmRNAs	1	1	1	1	1	1	1	1	1	1	1
CRISPR repeats	4	0	0	9	3	5	2	3	6	2	9
BioProject genome	PRJNA1082235	PRJNA1081642	PRJNA1081645	PRJNA523110	PRJNA1081640	PRJNA1081641	PRJNA310725	PRJNA205246	PRJNA310710	PRJNA310713	PRJDB6491
BioSample genome	SAMN40201554	SAMN40183415	SAMN40183414	SAMN10963686	SAMN40182996	SAMN40183110	SAMN04455154	SAMN02469826	SAMN04455122	SAMN04455153	SAMD00106843
Sequence Read Archive Illumina genome	SRR28210317	SRR28149488	SRR28161548	SRR9822007	SRR28148131	SRR28148136	SRR28314578	SRR28327341	SRR28147534	SRR28762162	–
Sequence Read Archive Nanopore genome	SRR28210318	SRR28161377	SRR28161549	SRR9822008	SRR28148125	SRR28149486	SRR28314177	SRR28327318	SRR28148124	SRR28762299	–
Assembly accession	CP155570	CP155572	CP156926-CP156927	CP036259-CP036260	CP155574	CP156925	CP146991-CP146992	CP146301	CP155571	CP155573	AP018449
GenBank 16S rRNA	PP693464	–	–	–	–	–	–	–	–	–	–

* This study.

† [62].

‡ [63].

§ [64].

**Fig. 1.**
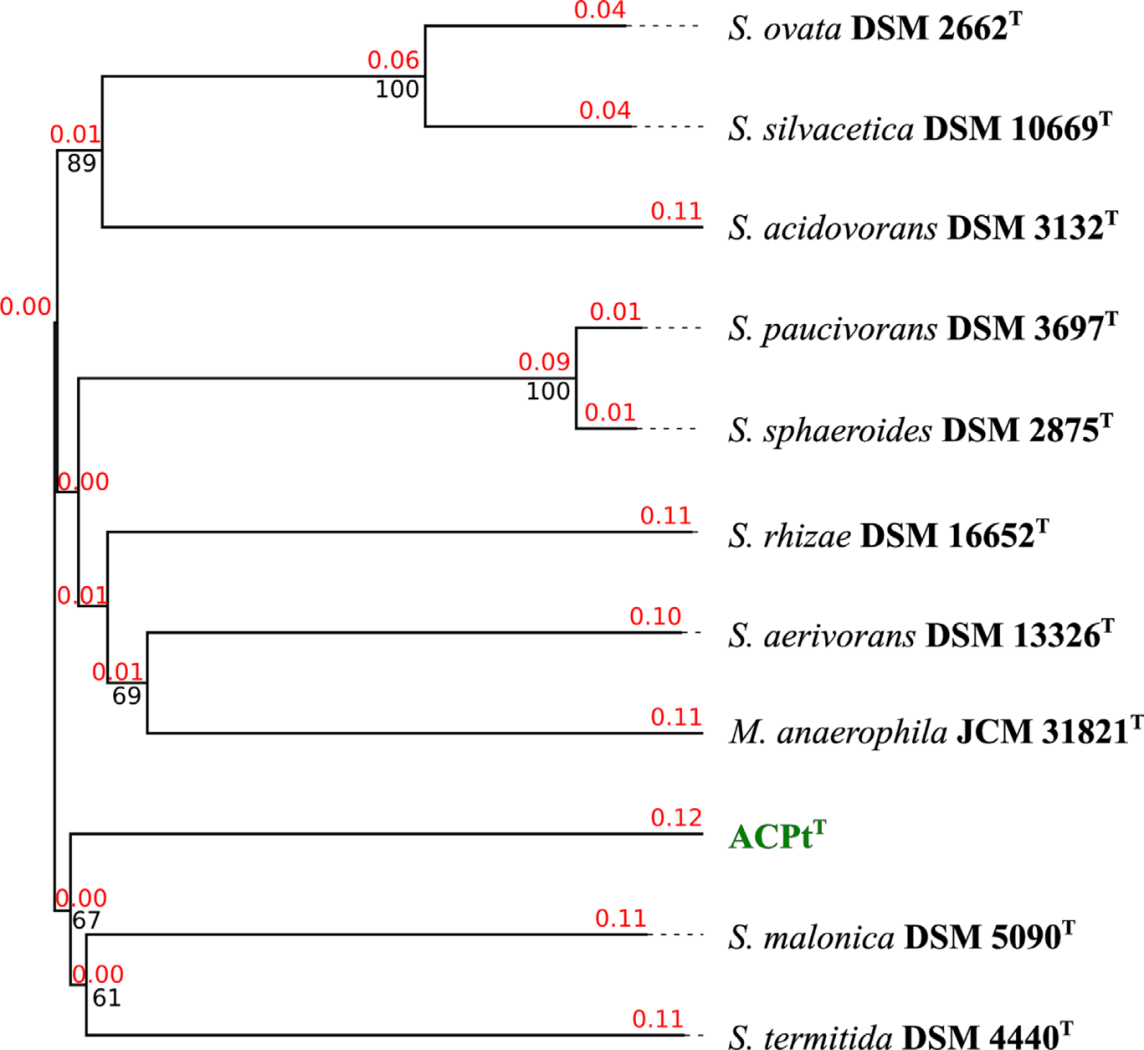
Whole-genome sequence-based phylogram of the novel ACPt^T^ isolate and the type strains of the genera *Sporomusa* and *Methylomusa*. Branch lengths are scaled in terms of GBDP distance formula *d*_5_. The red numbers above branches are GBDP pseudo-bootstrap support values >60% from 100 replications, with an average branch support of 71.5%. The black numbers below branches are confidence scores. The tree was rooted at the midpoint.

Data for the phylogenomic classification of the ACPt^T^ isolate to type strains of the genus *Sporomusa* and *Methylomusa* are summarized in Table 2. The phylogenomic comparison of strain ACPt^T^ with *Sporomusa* type strains and *M. anaerophila* showed orthoANI values ranging from 70.9 to 76.3% and dDDH (*d*_4_) [52] values ranging from 20.5 to 21.4%. As these values fell below the species threshold of under 95% for ANI and 70% for dDDH [41,53], the ACPt^T^ isolate represented a novel species in the genus *Sporomusa*. In order to determine whether the ACPt^T^ isolate could represent a novel genus in the family of *Sporomusaceae*, we additionally calculated POCP and AAI values. POCP values ranged from 56.4 to 63.3% and AAI values from 71.3 to 80.1%. POCP values of under 50% [44] and AAI values of 76% [54] were recommended as thresholds for genus delineation. In all AAI comparisons of the ACPt^T^ genome to *Sporomusa* reference genomes, we obtained values directly below and over the recommended genus threshold of 76%. The AAI comparison to the genome of *M. anaerophila* was 71.3% considerably lower than the threshold of 76%. This indicated that strain ACPt^T^ should be classified as a novel species of the genus *Sporomusa*. POCP comparisons of genomes from the genus *Sporomusa* yielded values considerably higher than the recommended 50% threshold for genus delineation. However, with a POCP value of 56.4%, this also applied to the comparison of strain ACPt^T^ with the *M. anaerophila* genome. This indicated that the 50% POCP threshold for genus delineation was not applicable to this phylogenomic cluster. The most closely related *Sporomusa* species was *S. malonica* with a POCP value of 63.3% and an AAI value of 80.1%. Correspondingly, based on the complete phylogenomic analysis, strain ACPt^T^ was classified as a novel species in the genus *Sporomusa* and the name *Sporomusa carbonis* is proposed. The whole-genome-based phylogram placed the genome of strain ACPt^T^ into a phylogenomic group with the type strains of *S. malonica* and *S. termitida* (Fig. 1) and the 16S rDNA gene sequence-based phylogram placed the ACPt^T^ isolate into a phylogenetic group with *S. aerivorans* (Fig. S1, available in the online Supplementary Material). The MLSA-based phylogram identified *S. malonica* as the most closely related *Sporomusa* species (Fig. S2).

**Table 2.** Phylogenomic analysis of strain ACPt^T^, the *Sporomusa* type strains of *S. malonica*, *S. aerivorans*, *S. termitida*, *S. paucivorans*, *S. rhizae*, *S. sphaeroides*, *S. ovata*, *S. acidovorans*, *S. silvacetica* and the type strain *M. anaerophila*

	orthoANI (%)	dDDH (*d*_4_)	POCP (%)	AAI (%)
*S. malonica*	76.3	21.4	63.3	80.1
*S. aerivorans*	74.2	20.7	58.0	77.3
*S. termitida*	74.3	20.9	61.8	77.2
*S. paucivorans*	74.7	21.2	63.5	76.8
*S. rhizae*	73.8	20.5	58.7	76.7
*S. sphaeroides*	74.9	21.0	61.8	76.5
*S. ovata*	74.0	21.2	59.1	75.9
*S. acidovorans*	73.5	20.2	61.0	75.8
*S. silvacetica*	73.9	21.2	57.4	75.7
*M. anaerophila*	70.9	21.1	56.4	71.3

**Fig. 2.**
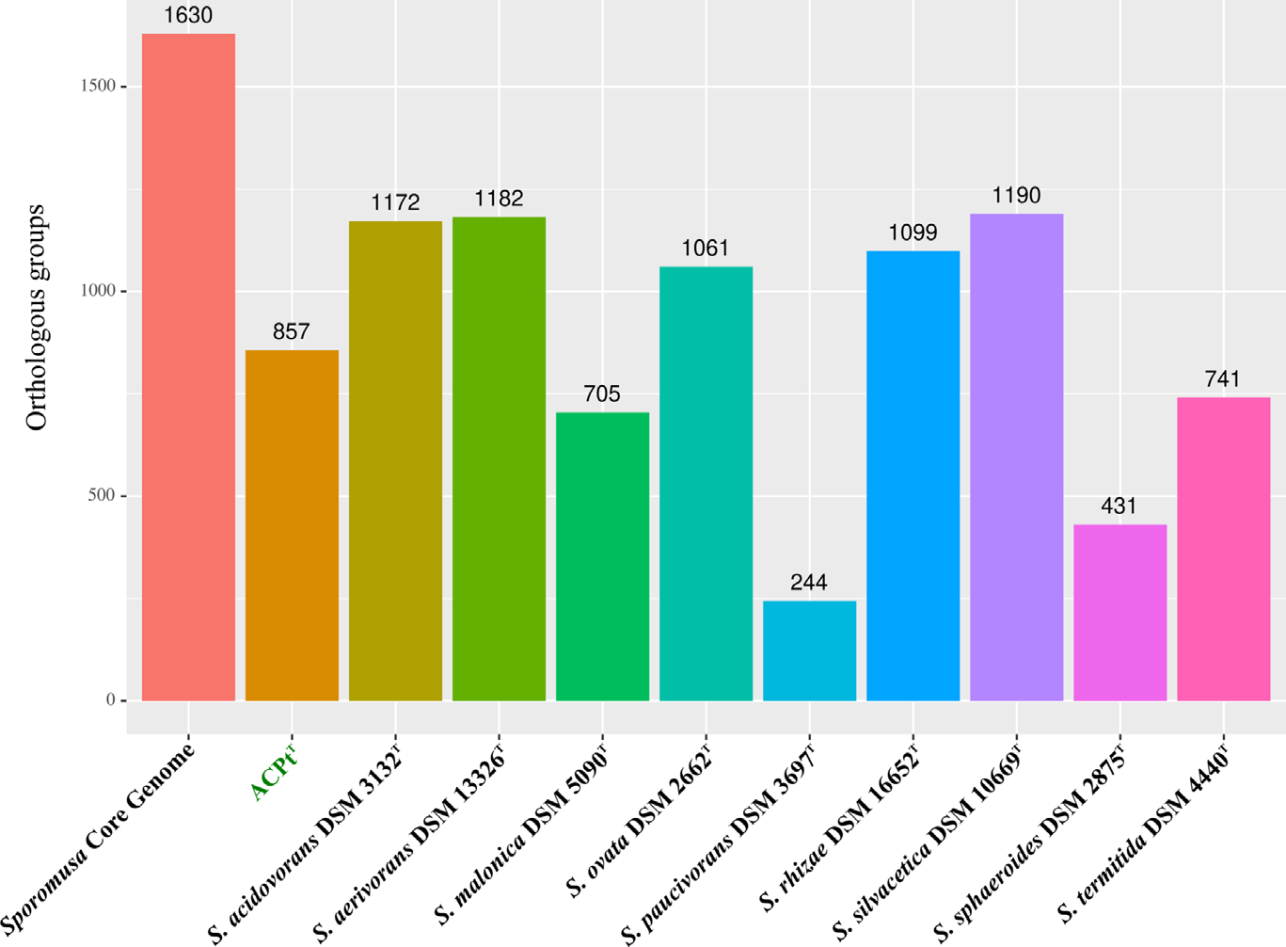
Shared orthologous groups between all *Sporomusa* type strains forming the *Sporomusa* core-genome and unique orthologous groups identified for every *Sporomusa type strain*.

**Fig. 3.**
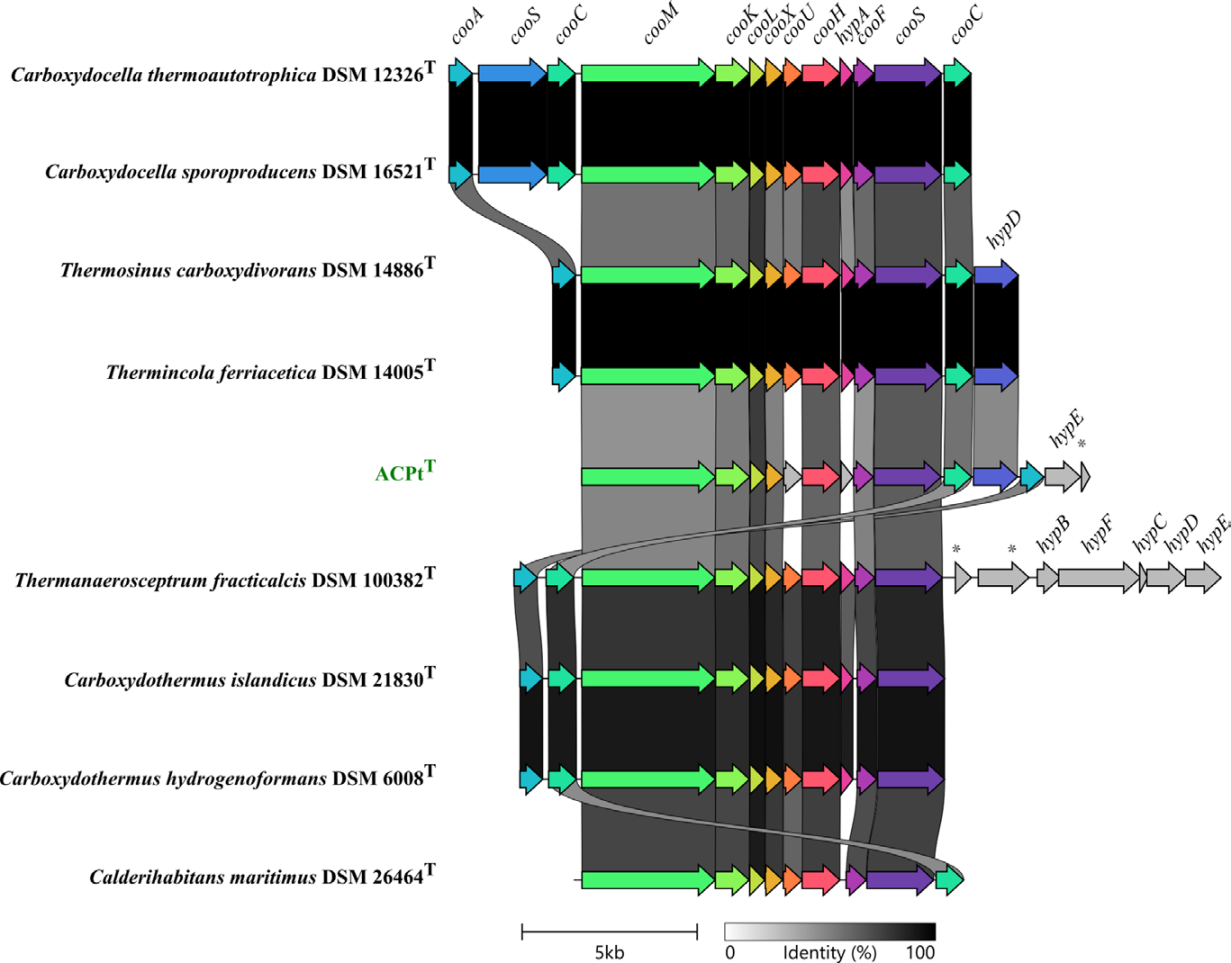
Structure of the gene cluster of the monofunctional carbon monoxide dehydrogenase in strain ACPt^T^ in comparison to carboxydotrophic hydrogenogens of thermophiles. Genes encoding proteins with 50% sequence identity or higher were given the same colour. The following gene abbreviations were used: *cooA*, transcriptional regulator CooA; *cooS*, monofunctional carbon monoxide dehydrogenase CooS; *cooC*, carbon monoxide dehydrogenase accessory protein; *cooM*, carbon monoxide dehydrogenase complex subunit CooM; *cooK*, carbon monoxide dehydrogenase complex subunit CooK; *cooL*, carbon monoxide dehydrogenase complex subunit CooL; *cooX*, carbon monoxide dehydrogenase complex subunit CooX; *cooU*, carbon monoxide dehydrogenase complex subunit CooU; *cooH*, carbon monoxide dehydrogenase complex subunit CooH; hypA, hydrogenase maturation factor HypA; *cooF*, ferredoxin type subunit of carbon monoxide dehydrogenase complex; *hypD*, hydrogenase maturation factor HypD; *hypE*, carbamoyl dehydratase HypE; *, hypothetical protein; *hypB*, hydrogenase maturation factor HypB; *hypF*, carbamoyltransferase HypF; *hypC*, hydrogenase maturation factor HypC.

Core/pan-genome analysis of the genus *Sporomusa* identified a pan-genome of 16 103 OGs and a core genome of 1630 OGs. The highest amount of unique OGs were identified for *S. silvacetica* (1190), *S. aerivorans* (1182), *S. acidovorans* (1172), * S. rhizae* (1,099) and *S. ovata* (1061), while lower amount of unique OGs were identified for strain ACPt^T^ (857), *S. termitida* (741), *S. malonica* (705), *S. sphaeroides* (431) and *S. paucivorans* (244) (Fig. 2). Unique OGs identified in the genome of strain ACPt^T^ comprised, for instance, a long-chain primary alcohol dehydrogenase (AdhA, SCACP_29740), a ferredoxin–NADP reductase (Fpr, SCACP_12760), a ferredoxin (Fer, SCACP_12770), a bifunctional homocysteine S-methyltransferase/5,10-methylenetetrahydrofolate reductase (YitJ, SCACP_12780), a pyruvate carboxylase (PycA, SCACP_22850) and a gene cluster encoding subunits of a CO-oxidizing/H_2_-evolving enzyme complex (CooMKLXUHFSC, SCACP_37230–37350). The latter gene cluster was most similar to the gene clusters of carboxydotrophic hydrogenogens from the thermophilic genera *Thermincola*, *Thermoanaerosceptrum, Thermosinus, Carboxydocella, Carboxydothermus* and *Calderihabitans* (Fig. 3).

All *Sporomusa* genomes encoded an Rnf complex, the WLP-gene cluster, a monofunctional carbon monoxide dehydrogenase (CooS), cytochromes b and c, ubiquinones, an HdrABC–MvhD complex (Hdr) and a FixAB complex (Fix). The Stn was encoded by the genomes of *S. ovata*, *S. silvacetica* and *M. anaerophila*, while other *Sporomusa* species harboured instead genes coding for a three-subunit electron-bifurcating hydrogenase (HydABC) at this genome location. *Sporomusa* species lacking the Stn transhydrogenase contained genes for the Nfn transhydrogenase [15,55]. An overview of the detected enzymes and complexes potentially involved in lithotrophic growth of the genus *Sporomusa* is shown in Table 3.

**Table 3.** Genomic analysis of the type strains of the genera *Sporomusa* and *Methylomusa* with regard to the presence of the Rnf complex (Rnf), Ech complex (Ech), WLP, monofunctional carbon monoxide dehydrogenase (CooS), cytochromes (Cyt), ubiquinones (UQ), HdrABC-MvhD complex (Hdr), FixAB complex (Fix), NfnAB transhydrogenase (Nfn) and StnABC transhydrogenase (Stn)

	Rnf	Ech	WLP	CooS	Cyt B/c	UQ	Hdr	Fix	Nfn	Stn
ACPt^T^	+	+	+	+	+	+	+	+	+	−
*S. malonica*	+	−	+	+	+	+	+	+	+	−
*S. aerivorans*	+	−	+	+	+	+	+	+	+	−
*S. termitida*	+	−	+	+	+	+	+	+	+	−
*S. paucivorans*	+	−	+	+	+	+	+	+	+	−
*S. rhizae*	+	−	+	+	+	+	+	+	+	−
*S. sphaeroides*	+	−	+	+	+	+	+	+	+	−
*S. ovata*	+	*	+	+	+	+	+	+	−	+
*S. acidovorans*	+	−	+	+	+	+	+	+	+	−
*S. silvacetica*	+	−	+	+	+	+	+	+	−	+
*M. anaerophila*	+	−	+	+	+	+	+	−	−	+

+, Gene cluster present; −, gene cluster absent.

* Ech-like complex, not in synteny of *cooS*.

**Fig. 4.**
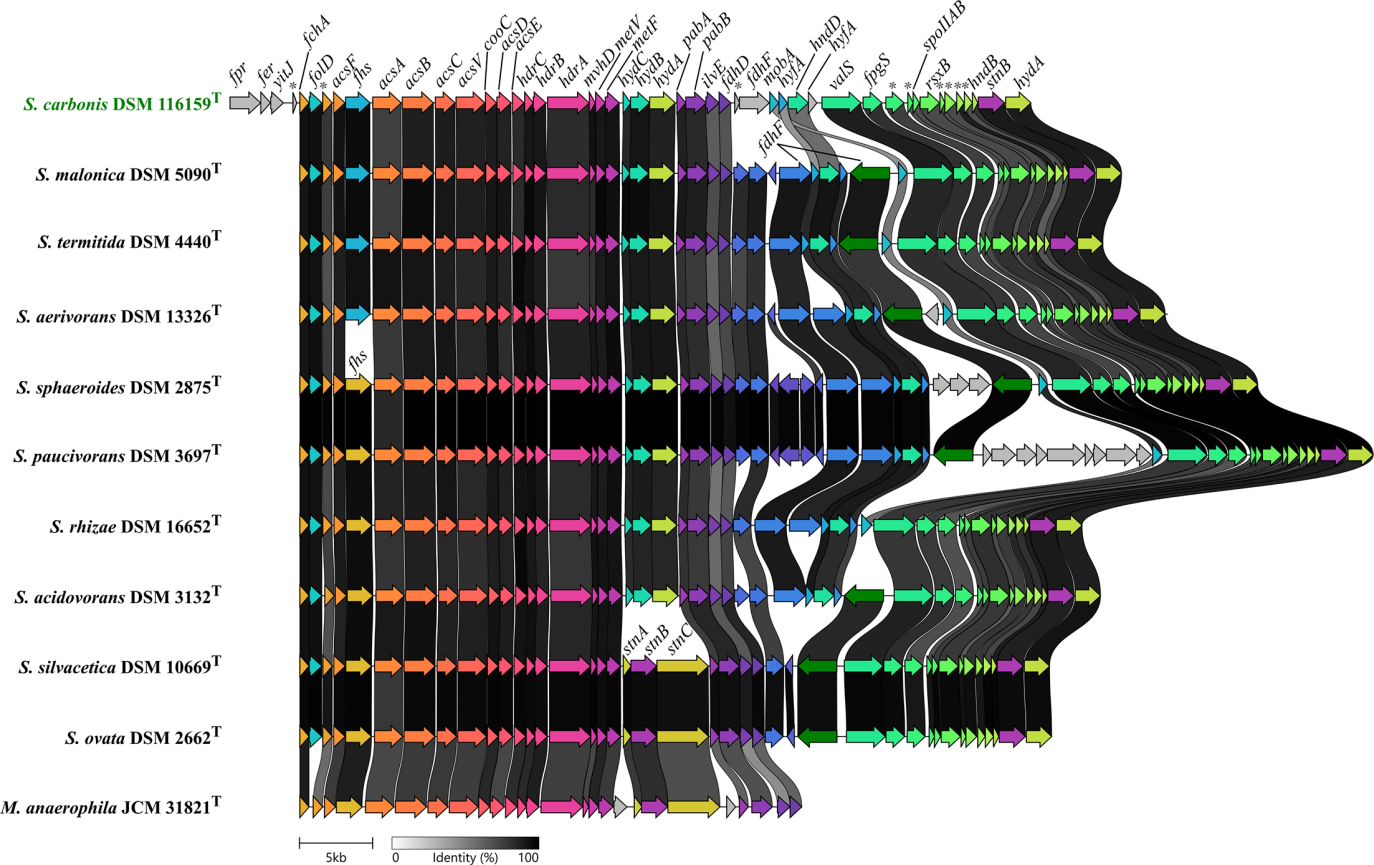
Structure of the Wood–Ljungdahl gene cluster in type strains of the genus *Sporomusa* and *Methylomusa*. Genes encoding proteins with 50% sequence identity or higher were given the same colour. The following gene abbreviations were used: *fpr*, ferredoxin–NADP reductase; *fer*, ferredoxin ;*yitJ*, bifunctional homocysteine *S*-methyltransferase/5,10-methylenetetrahydrofolate reductase; *, hypothetical protein; *fchA*, methenyl THF cyclohydrolase; *folD*, bifunctional cyclohydrolase/dehydrogenase; *acsF*, carbon monoxide dehydrogenase accessory protein; *fhs*, formyl THF synthetase; *acsA*, anaerobic carbon-monoxide dehydrogenase catalytic subunit; *acsB*, carbon monoxide dehydrogenase/acetyl-CoA synthase subunit beta; *acsC*, CoFeSP large subunit; *acsV*, corrinoid activation/regeneration protein; *cooC*, carbon monoxide dehydrogenase accessory protein; *acsD*, CoFeSP small subunit; *acsE*, methyl THF CoFeSP methyltransferase; *hdrC*, heterodisulfide oxidoreductase iron–sulphur cluster-binding subunit; *hdrB*, heterodisulfide reductase subunit B; *hdrA*, heterodisulfide reductase subunit A; *mvhD*, methyl-viologen-reducing hydrogenase delta subunit; *metV*, methylene THF reductase C-terminal catalytic subunit; *metF*, methylene THF reductase large subunit; *hydC*, electron bifurcating hydrogenase subunit HydC; *hydB*, electron bifurcating hydrogenase subunit HydB; *hydA*, electron bifurcating hydrogenase subunit HydA; *stnA*, Stn subunit A; *stnB*, Stn subunit B; *stnC*, Stn subunit C; *pabA*, aminodeoxychorismate/anthranilate synthase component 2; *pabB*, aminodeoxychorismate synthase component 1; *ilvE*, branched-chain-amino-acid aminotransferase; *fdhD*, sulphur carrier protein; *fdhF*, formate dehydrogenase H; *mobA*, molybdenum cofactor guanylyltransferase; *hyfA*, hydrogenase-4 component A; *valS*, valine-tRNA ligase; *fpgS*, folylpolyglutamate synthase; *spoIIAB*, anti-sigma F factor; *rsxB*, ion-translocating oxidoreductase complex subunit B; *hndB*, NADP-reducing hydrogenase subunit HndB.

The structure of the genomic region encoding the WLP gene cluster and adjacent genes in the genus *Sporomusa* and *Methylomusa* is visualized in Fig. 4. The structure of the WLP gene cluster, including the genes encoding the Hdr complex, was highly conserved in *Sporomusa* and *Methylomusa* members. The genome of strain ACPt^T^ additionally encoded a ferredoxin–NADP reductase gene (*fpr*) in synteny with a ferredoxin (*fer*) and a bifunctional homocysteine S-methyltransferase/5,10-methylenetetrahydrofolate reductase (*yitJ*) upstream of the WLP gene cluster. The structure of the genes downstream of the Hdr complex in the *Sporomusa* genomes differed by the presence of the HydABC hydrogenase or the StnABC transhydrogenase. They also differed in the type and amount of formate dehydrogenases being encoded. All *Sporomusa* genomes coded for additional hydrogenase subunit genes (*hydA, stnB* and *hndB*) in synteny with several genes for hypothetical proteins at the end of this genomic region.

### Morphological, physiological and chemotaxonomic characterization

**Fig. 5.**
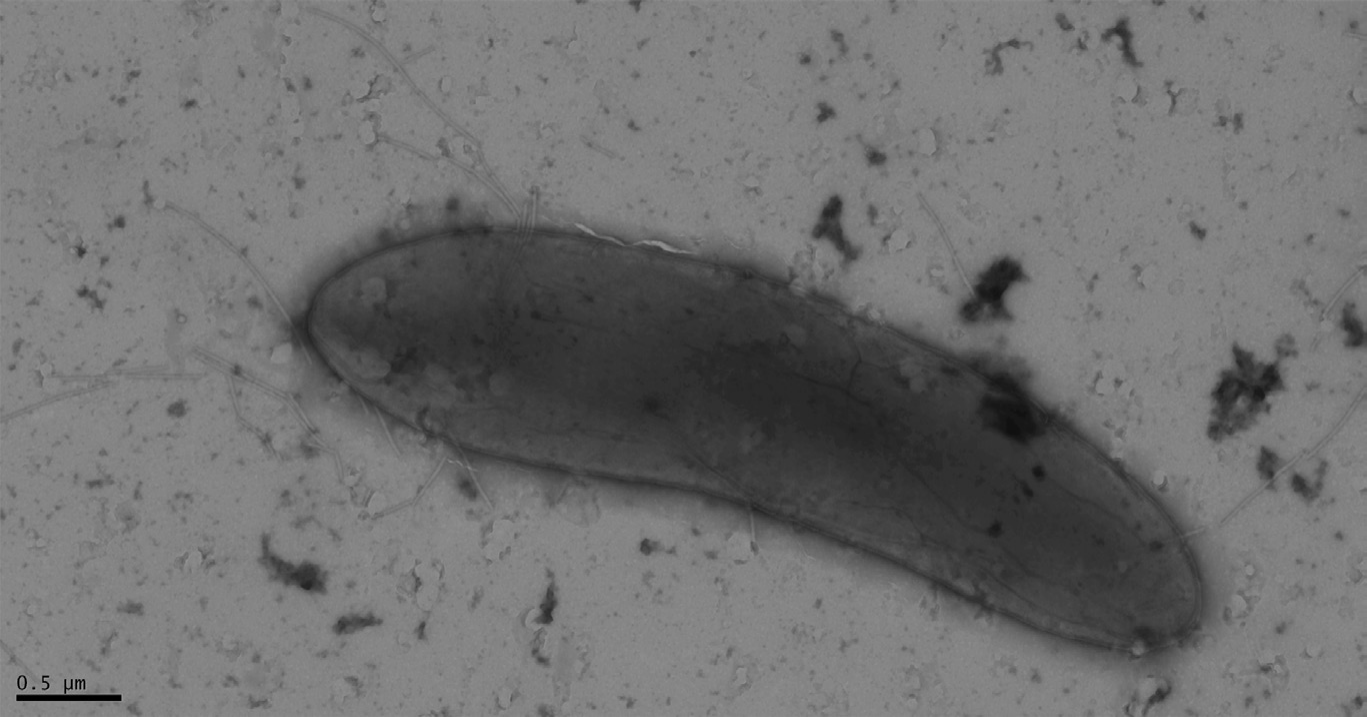
Transmission electron micrograph of a negatively stained cell of strain ACPt^T^.

An overview of the morphological and physiological characterization of strain ACPt^T^ and the reference data of other *Sporomusa* type strains is shown in Table 4. Cells of strain ACPt^T^ showed the *Sporomusa* characteristic curvature of the rod-shaped cells (Fig. 5), stained Gram-negative and showed growth only under strict anoxic conditions. Growth occurred in the temperature range of 35–50 °C and was optimal at 40 °C under the tested conditions. In comparison to the reported data for other *Sporomusa* species growing between 15 and 45 °C, the temperature range of strain ACPt^T^ was shifted towards higher temperatures. With growth occurring at 50 °C, strain ACPt^T^ showed the highest growth temperature reported for a *Sporomusa* type strain. Furthermore, strain ACPt^T^ grew optimally when NaCl was not added to the medium and at pH 7. The NaCl tolerance of other *Sporomusa* species has not been reported; the determined pH optimum of 7 was in line with the data of other *Sporomusa* species descriptions. Cellular fatty acid analysis identified Iso-βOH-C_13 : 0_ (15.6%), Iso-C_15 : 0_ (11.4%) and Iso-C_11 : 0_ (9.0%) as the predominant cellular fatty acids of strain ACPt^T^. In comparison to the reported cellular fatty acid profile of *S. ovata* An4, *S. ovata* DSM 2662^T^ and *S. aerivorans* DSM 13326^T^ [11], strain ACPt^T^ contained higher amounts of C_15 : 0_, Iso-C_15 : 0_ and lower amounts of C_16 : 1_ ∆7 (Table S1). Strain ACPt^T^ utilized ribose, fructose, glucose, sucrose, lactose, melezitose, pyruvate, vanillate, syringate, methanol and CO for growth. Growth with glucose, sucrose, lactose and melezitose was not reported for any other *Sporomusa* species and was thereby identified as a potential unique metabolic feature of strain ACPt^T^. Arabinose, xylose, galactose, mannose, rhamnose, glucuronic acid, glycerol, sorbitol, mannitol, inositol, cellobiose, trehalose, maltose, melibiose, raffinose, formate, lactate, malate, 3-OH-butyrate, citrate, sarcosine, DMG, betaine, ethanol, propanol, 1,2-propanediol, butanol and H_2_+CO_2_ were not utilized for growth under the tested conditions. Resting cell experiments showed the production of H_2_ from CO but not the production of acetate from H_2_+CO_2_ under the tested conditions. These observations imply that strain ACPt^T^ employs the metabolism of a carboxydotrophic hydrogenogen, transforming CO with the water–gas shift reaction to H_2_+CO_2_, which is coupled to energy conservation at a membrane-associated Ech complex [56]. This hypothesis was further supported by the identification of the strain ACPt^T^ unique *cooMKLXUHFSC* gene cluster during the pan/core-genome analysis of the genus *Sporomusa*. The metabolism of carboxydotrophic hydrogenogens was hypothesized to detoxify CO-exposed microenvironments [57]. In the charcoal-burning pile environment, strain ACPt^T^ was exposed to high concentrations of CO, which putatively led to the adaptation of an acetogenic bacterium to a carboxydotrophic hydrogenogen for the advantage of rapid detoxification. The *cooMKLXUHFSC* gene cluster was hypothesized to be propagated by horizontal gene transfer, as this genomic region shows high similarities between isolated strains of phylogenetically diverse taxa [57]. The inability of strain ACPt^T^ to utilize H_2_+CO_2_ for growth despite possessing the required gene sets for acetogenesis matches the description of other carboxydotrophic hydrogenogens encoding a complete WLP [58,59]. The major putative function of the WLP in carboxydotrophic hydrogenogens is the CO_2_-based production of acetyl-CoA for the generation of biomass fuelled by the oxidation of CO [56,60]. However, the WLP was shown to also be able to function as a backup metabolism in carboxydotrophic hydrogenogens enabling acetogenic growth. For example, the carboxydotrophic hydrogenogen *Carboxydothermus hydrogenoformans* was shown to switch to acetogenic growth upon sufficient depletion of CO and partial pressure increase of H_2_+CO_2_ [61]. Correspondingly, we cannot exclude the possibility that under specific conditions strain ACPt^T^ is able to utilize H_2_+CO_2_ for growth by acetogenesis.

**Table 4.** Morphological and physiological characterization of strain ACPt^T^ in comparison to the data reported for other *Sporomusa* type strains

Substrate	ACPt^T^	S. malonica*	*S. aerivorans*†	*S. termitida*‡	*S. paucivorans*§	*S. rhizae*¶	S. sphaeroides**	S. ovata**	*S. acidovorans*††	*S. silvacetica*‡‡	*M. anaerophila*§§
Gram staining	Negative	Negative	Negative	Negative	Negative	Negative	Negative	Negative	Negative	Negative	Negative
Temperature optimum	40 °C	28–32 °C	30 °C	30 °C	nr	35 °C	35–39 °C	34–39 °C	35 °C	30 °C	nr
Temperature range	35–50 °C	15–38 °C	19–35 °C	19–37 °C	nr	15–40 °C	15–45 °C	15–45 °C	20–40 °C	10–35 °C	30–37 °C
NaCl optimum	0%	nr	nr	nr	nr	nr	nr	nr	nr	nr	nr
NaCl range	0–1%	nr	nr	nr	nr	nr	nr	nr	nr	nr	nr
pH optimum	7.0	7.3	7	7.2	nr	7.5	6.4–7.6	5.3–7.2	6.5	6.8	5.9–6.9
L(+)-Arabinose	−	−	nr	−	−	nr	−	−	nr	−	−
D(-)-Ribose	+	nr	nr	−	−	nr	−	−	+	nr	nr
D(+)-Xylose	−	−	nr	−	−	−	−	−	nr	−	−
D(-)-Fructose	+	+	−	−	−	−	−	+	+	+	−
D(+)-Galactose	−	nr	nr	−	−	nr	−	−	nr	nr	nr
D(+)-Glucose	+	−	−	−	−	−	−	−	nr	−	−
D(+)-Mannose	−	nr	nr	−	−	nr	−	−	nr	−	−
L(+)-Rhamnose	−	nr	nr	nr	−	nr	−	−	nr	−	−
D(-)-Glucuronic acid	−	nr	nr	nr	−	nr	nr	nr	nr	nr	nr
Glycerol	−	−	−	−	+	nr	+	−	+	+	−
D(-)-Sorbitol	−	nr	nr	−	nr	nr	−	−	nr	−	−
D(-)-Mannitol	−	nr	+	+	−	nr	−	−	nr	−	−
myo-Inositol	−	nr	nr	nr	−	nr	−	−	nr	nr	nr
D(+)-Sucrose	+	nr	nr	−	−	−	−	−	nr	−	−
D(+)-Cellobiose	−	nr	−	−	−	nr	−	−	nr	−	−
D(+)-Trehalose	−	nr	−	−	−	nr	−	−	nr	−	−
D(+)-Lactose	+	−	−	−	−	nr	−	−	nr	−	−
D(+)-Maltose	−	nr	nr	−	−	nr	−	−	nr	−	−
D(+)-Melibiose	−	nr	nr	−	nr	nr	−	−	nr	nr	nr
D(+)-Raffinose	−	nr	nr	−	−	−	−	−	nr	−	−
D(+)-Melezitose	+	nr	nr	nr	−	nr	−	−	nr	−	−
Formate	−	+	+	+	+	+	+	+	+	+	nr
Pyruvate	+	+	+	+	+	nr	+	+	+	+	+
dl-Lactate	−	+	+	+	+	+	+	+	−	+	+
dl-Malate	−	+	+	−	−	nr	−	−	+	nr	nr
3-OH-butyrate	−	+	nr	nr	nr	nr	+	−	nr	nr	nr
Citrate	−	+	+	+	−	+	−	−	nr	−	nr
Sarcosine	−	nr	nr	+	−	nr	+	+	nr	nr	−
DMG	−	nr	nr	−	−	nr	+	+	nr	nr	−
Betaine	−	+	nr	+	+	+	+	+	nr	+	−
Vanillate	+	nr	+	nr	nr	+	nr	nr	nr	+	nr
Syringate	+	nr	+	nr	nr	+	nr	nr	nr	nr	nr
Methanol	+	+	+	+	+	nr	+	+	+	+	+
Ethanol	−	+	+	+	+	−	+	+	−	+	−
n-Propanol	−	+	nr	−	+	nr	+	+	nr	nr	nr
1,2-Propanediol	−	+	nr	nr	+	nr	+	+	nr	nr	nr
n-Butanol	−	+	nr	nr	+	nr	+	+	nr	nr	−
H_2_/CO_2_	−	+	+	+	+	+	+	+	+	+	−
CO (product)	+ (H_2_+CO_2_)	nr	nr	+ (nr)	nr	−	nr	+ (acetate)	nr	−	nr

+, Positive; −, negative; nr, not reported.

* Data from [5].

† Data from [7].

‡ Data from [3].

§ Data from [2].

¶ Data from [8].

** Data from [1,26].

†† Data from [4].

‡‡ Data from [6].

§§ Data from [64].

## Description of *Sporomusa carbonis* sp. nov.

*Sporomusa carbonis* (car.bo’nis. L. gen. n. *carbonis*, of coal, of charcoal). Cells show the form of a curved rod and stain Gram-negative. Growth occurs only under strict anoxic conditions and ranges from 35 to 50 °C (optimum 40 °C) and added NaCl concentrations from 0 to 1% and optimal without addition. The optimal pH for growth is 7, and maximal growth occurs with glucose as a substrate. Furthermore, the substrates ribose, fructose, glucose, sucrose, lactose, melezitose, pyruvate, vanillate, syringate and methanol are utilized. CO is transformed into H_2_ and CO_2_ as the major fermentation products. It does not grow on H_2_+CO_2_, arabinose, xylose, galactose, mannose, rhamnose, glucuronic acid, glycerol, sorbitol, mannitol, inositol, cellobiose, trehalose, maltose, melibiose, formate, lactate, malate, 3-OH-butyrate, citrate, sarcosine, DMG, betaine, ethanol, propanol, 1,2-propanediol and butanol. The predominant cellular fatty acids are Iso-βOH-C_13 : 0_ (15.6%), Iso-C_15 : 0_ (11.4%) and Iso-C_11 : 0_ (9.0%). The type strain genome comprises 4.1 Mbp with a G+C content of 46.2 mol% and is available under the GenBank/EMBL/DDBJ accession number CP155570. The type strain ACPt^T^ was isolated from the top of the covering soil of an active charcoal burning pile, Hasselfelde, Germany. The GenBank/EMBL/DDBJ accession number for the 16S rRNA gene is PP693464. The type strain of *S. carbonis* is ACPt^T^ and was deposited under the identifiers DSM 116159^T^ and CCOS 2105^T^.

## Supplementary material

10.1099/ijsem.0.006677 Uncited Supplementary Material 1.

## Abbreviations

AAI: average amino acid identity
ANI: average nucleotide identity
dDDH: digital DNA–DNA hybridization
MLSA: multilocus sequence analysis
OGs: orthologous groups
POCP: percentage of conserved proteins
Stn: Sporomusa-type Nfn transhydrogenase
WLP: Wood–Ljungdahl pathway
