# Supplementary material for: Isolation and characterization of Sporomusa carbonis sp. nov.: a carboxydotrophic hydrogenogen in the genus of Sporomusa isolated from a charcoal-burning pile
Source: Int J Syst Evol Microbiol. 2025 Apr 16;75(4):006677. doi: 10.1099/ijsem.0.006677 (PMC12003927; doi:10.1099/ijsem.0.006677)
Supplement: Uncited Supplementary Material 1. [file ijsem-75-06677-s001.pdf]

Supplementary Material

Table S1. Cellular fatty acid composition of *S. carbonis* (strain ACPt^T) in comparison to the reported data (Balk et al. 2010) for *S. aerivorans* DSM 13326^T, *S. ovata* DSM 2662^T and *S. ovata* DSM 21435.

Fatty acid	*S. carbonis* DSM 116159^T	*S. aerivorans* DSM 13326^{T*}	*S. ovata* DSM 2662^{T*}	*S. ovata* DSM 21435*
$C_{11:0}$	0.5	-	-	-
Iso-$C_{11:0}$	9.0	1.1	1.2	2.3
Iso-$C_{11:0}$ DMA	0.2	-	-	-
Iso-βOH-$C_{11:0}$	0.3	-	-	-
βOH-$C_{11:0}$	-	0.9	1.3	-
βOH-$C_{12:0}$	1.4	10.5	7.3	1.5
$C_{13:0}$	0.3	-	-	-
$C_{13:1}$ Δ3	0.9	-	-	-
Iso-$C_{13:0}$	0.2	-	-	-
Iso-βOH-$C_{13:0}$	15.6	8.6	8.0	26.5
βOH-$C_{13:0}$	-	2.0	4.1	-
βOH-$C_{13:1}$	-	0.8	1.1	-
$C_{14:0}$	3.0	1.1	1.2	0.5
$C_{14:0}$ DMA/βOH-$C_{13:0}$/Iso-$C_{15:0}$ Δ7	1.3	-	-	-
$C_{14:1}$ Δ5	0.2	-	-	-
$C_{15:0}$	5.5	1.5	3.8	0.9
$C_{15:0}$ DMA	1.5	-	-	-
$C_{15:0}$ ALDE	0.1	-	-	-
Anteiso $C_{15:0}$	0.2	-	-	-
$C_{15:1}$ Δ7	-	7.3	11.6	1.3
Iso-$C_{15:0}$	11.4	0.8	0.7	4.3
Iso-$C_{15:0}$ DMA	0.4	-	-	-
Iso-$C_{15:1}$ Δ5	0.7	-	-	-
Iso-$C_{15:1}$ Δ5+7	2.3	-	-	-
Iso-$C_{15:1}$ Δ7	2.4	-	-	-
Iso-$C_{15:1}$ Δ9	0.3	-	-	-
Iso-$C_{15:1}$ Δ7+9	-	-	-	0.9
$C_{16:0}$	4.7	7.1	7.2	4.0
Iso-$C_{16:0}$	-	-	-	0.7
$C_{16:1}$ Δ7	5.2	27.5	20.6	7.0
$C_{16:1}$ Δ7 DMA	0.7	-	-	-
$C_{16:1}$ Δ? DMA	0.5	-	-	-
$C_{16:1}$ Δ9	3.6	4.0	2.2	1.9
$C_{16:1}$ Δ11	-	0.5	0.6	0.6
Iso-$C_{16:1}$ Δ7	-	-	0.4	0.4
$C_{17:0}$	0.3	0.7	1.7	0.5
$C_{17:0}$ DMA	0.3	-	-	-
$C_{17:0}$ cyclo Δ9/$C_{17:1}$ Δ? DMA	5.9	-	-	-
Anteiso-$C_{17:0}$	-	-	-	3.1
$C_{17:1}$ Δ7	-	2.7	6.1	2.0
$C_{17:1}$ Δ7/$C_{17:1}$ Δ9/$C_{16:0}$ DMA	2.6	-	-	-
$C_{17:1}$ Δ9	-	10.2	12.6	1.4
$C_{17:1}$ Δ7+9	2.4	-	-	-
$C_{17:1}$ Δ11	-	1.1	1.5	-
$C_{17:1}$ Δ? DMA	1.5	-	-	-

$C_{17:1}$ Δ? DMA	1.8	-	-	-
Iso-$C_{17:1}$	1.8	-	-	-
Iso-$C_{17:1}$ Δ7	-	2.0	1.7	22.3
Iso-$C_{17:1}$ Δ9	-	1.0	0.6	5.6
Iso-$C_{17:1}$ Δ7+9	8.5	-	-	-
$C_{18:0}$	0.3	0.7	0.6	1.2
$C_{18:1}$ Δ9	0.3	5.5	3.4	2.1
$C_{18:1}$ Δ11	0.8	2.5	1.0	0.5
Iso-$C_{19:1}$ Δ9	-	-	-	0.7

* data from [12]

DMA – dimethyl acetal

ALDE - aldehyde

Δ? – position of double-bond unclear

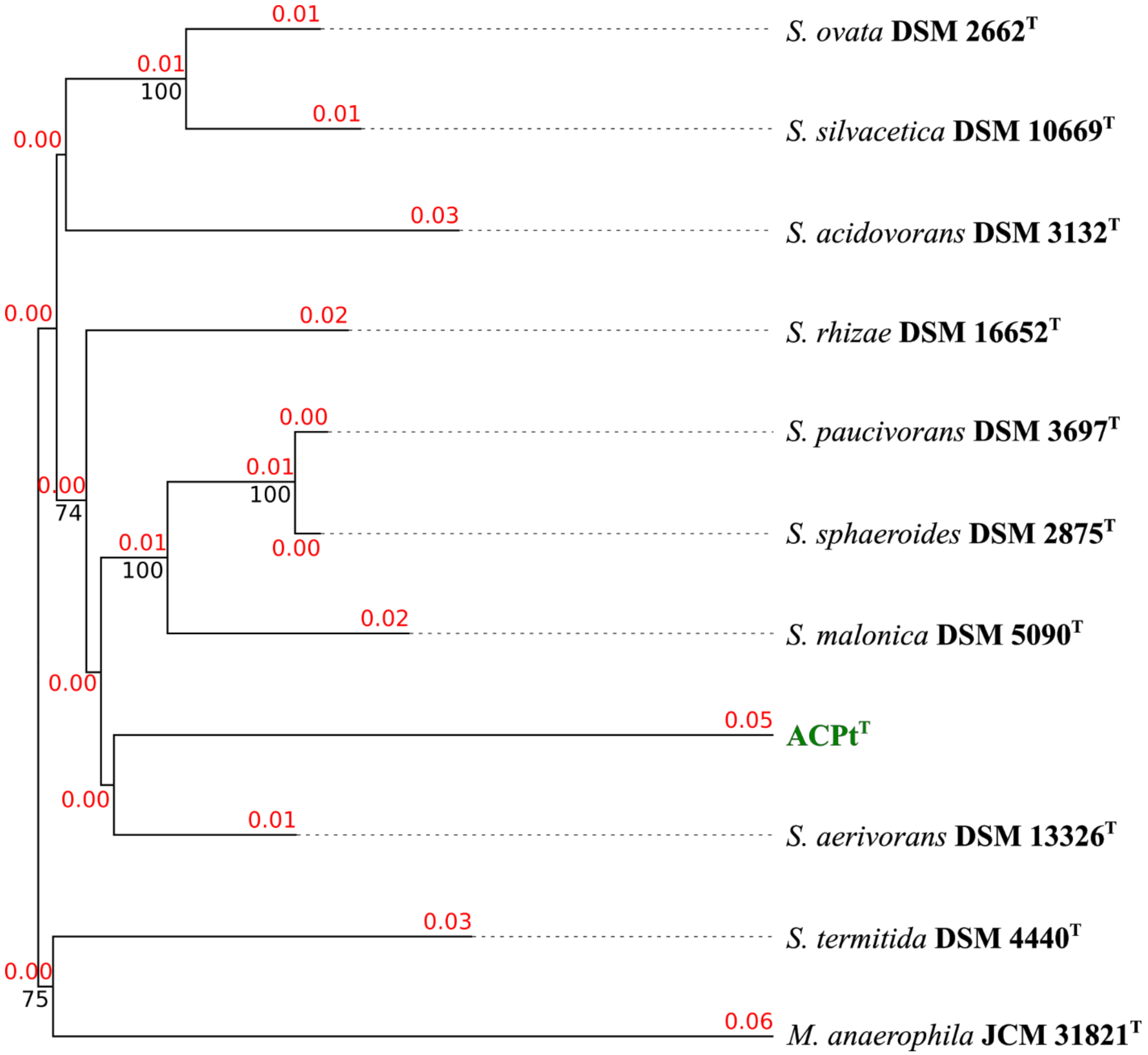

Figure S1. 16S rDNA gene sequence-based phylogram of the novel ACPt^T isolate and the type strains of the genera *Sporomusa* and *Methylomusa*. Branch lengths are scaled in terms of GBDP distance formula d5. Red numbers above branches are GBDP pseudo-bootstrap support values > 60% from 100 replications, with an average branch support of 73.1%. Black numbers below branches are confidence scores. The tree was rooted at the midpoint.

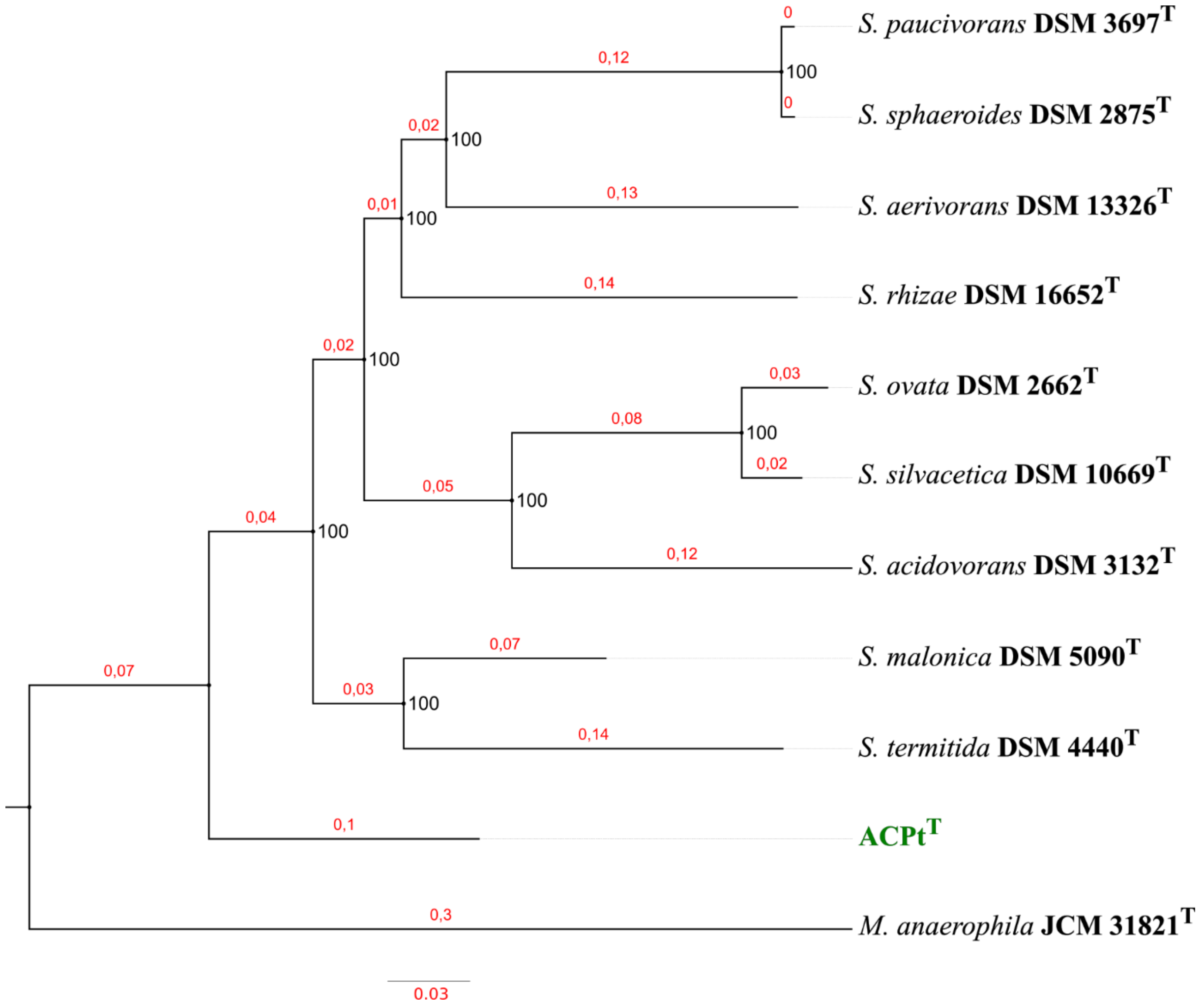

Fig S2. MLSA-based phylogram of amino acid sequences derived from orthologues (1350 genes concatenated to a combined aligned length of 404,654 residues per organism) of the novel ACPt^{T} isolate and the type strains of the genera *Sporomusa* and *Methylomusa*. Red numbers depict branch lengths derived by RAxML (Randomized Axelerated Maximum Likelihood). Black numbers next to branches are confidence scores. The tree was visualized with Figtree, rooted at the midpoint and nodes were sorted in increasing order.
